# Suppression of Circadian Timing and Its Impact on the Hippocampus

**DOI:** 10.3389/fnins.2021.642376

**Published:** 2021-04-08

**Authors:** Norman F. Ruby

**Affiliations:** Biology Department, Stanford University, Stanford, CA, United States

**Keywords:** Siberian, hamster, theta, acetylcholine, dentate gyrus, memory

## Abstract

In this article, I describe the development of the disruptive phase shift (DPS) protocol and its utility for studying how circadian dysfunction impacts memory processing in the hippocampus. The suprachiasmatic nucleus (SCN) of the Siberian hamster is a labile circadian pacemaker that is easily rendered arrhythmic (ARR) by a simple manipulation of ambient lighting. The DPS protocol uses room lighting to administer a phase-advancing signal followed by a phase-delaying signal within one circadian cycle to suppress clock gene rhythms in the SCN. The main advantage of this model for inducing arrhythmia is that the DPS protocol is non-invasive; circadian rhythms are eliminated while leaving the animals neurologically and genetically intact. In the area of learning and memory, DPS arrhythmia produces much different results than arrhythmia by surgical ablation of the SCN. As I show, SCN ablation has little to no effect on memory. By contrast, DPS hamsters have an intact, but arrhythmic, SCN which produces severe deficits in memory tasks that are accompanied by fragmentation of electroencephalographic theta oscillations, increased synaptic inhibition in hippocampal circuits, and diminished responsiveness to cholinergic signaling in the dentate gyrus of the hippocampus. The studies reviewed here show that DPS hamsters are a promising model for translational studies of adult onset circadian dysfunction in humans.

## Introduction

The formal properties of the circadian system of Siberian hamsters (a.k.a., Djungarian hamsters; *Phodopus sungorus*) have been thoroughly characterized over the last 50 years, mainly as a model species for studies of melatonin signaling of daylength (Bartness et al., 1993). Over those years, the circadian system of these hamsters has been evaluated for its responses to photic signals in a range of photoperiod studies, entrainment paradigms, and phase responses, all of which showed that circadian responses in this species are similar to those observed in other nocturnal rodents. And yet, when these animals are challenged by a photic phase-advancing stimulus, followed by a photic phase-delaying stimulus less than 24 h later, the opposing light signals completely eliminate circadian timing in the molecular clock in the suprachiasmatic nucleus (SCN) within just a few days.

The unique vulnerability to rhythm disruption in an otherwise typical circadian system suggests that there is a great deal more to learn about photic effects on circadian timing, but it also presents an opportunity to study the adverse impact of circadian disruption in a new way. Animal models of circadian arrhythmia have been limited in their relevance to human circadian disruption because they involve surgical ablation of the SCN or genomic modifications that are obviously not present in humans, and because they have confounding effects on sleep and stress. By contrast, the circadian-arrhythmic (ARR) hamster lacks these confounds (cf., Larkin et al., 2004), thus allowing us to better model how a malfunctioning SCN affects the progression of human illnesses. The circadian-ARR hamster model has revealed how the loss of circadian timing impairs different types of memory (Fernandez et al., 2014; Müller et al., 2015), immune responses (Prendergast et al., 2013), and has furthered our understanding of sleep homeostasis (Larkin et al., 2004) and ultradian rhythm generation (Prendergast et al., 2012). This article presents the development of the disruptive phase shift (DPS) protocol, and shows how it is useful for modeling circadian influences on hippocampal memory processing, an issue that has become important recently in elderly populations (Tranah et al., 2011; Covell et al., 2012; Coogan et al., 2013).

## Development of the Dps Model: Chance Favors the Distracted Mind

**FIGURE 1:**
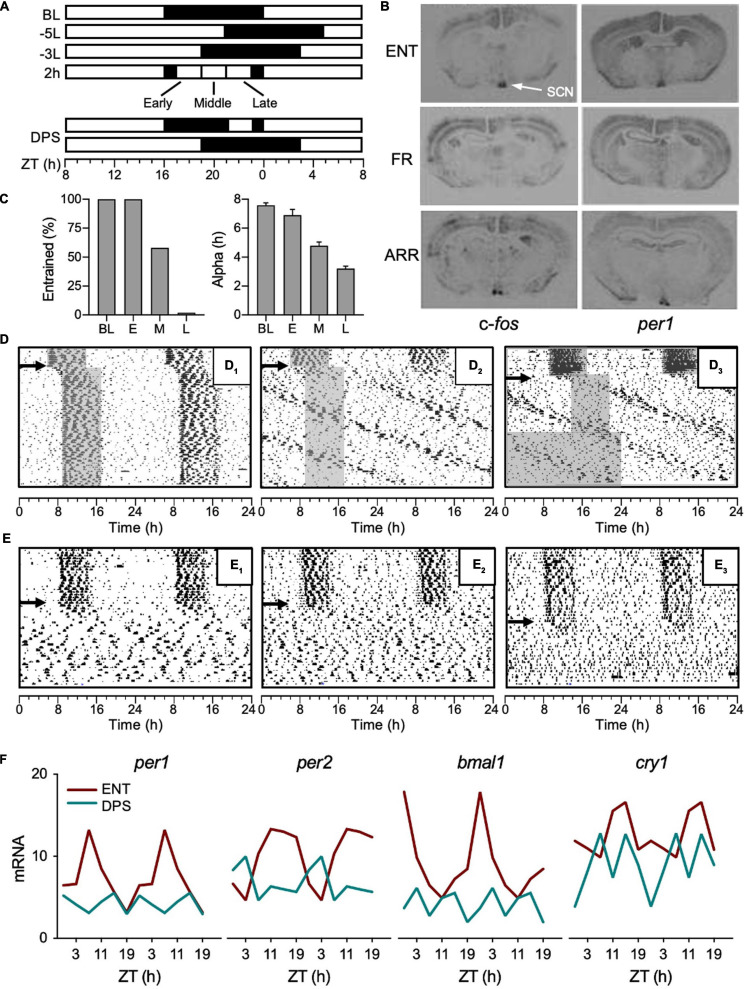
Development of the DPS protocol. **(A)** Light-dark (LD) cycles given by white and black rectangles, respectively. A phase advance or delay of the LD cycle is indicated by a plus (+) or min (–) sign, respectively, followed by phase shift duration in hours; L or D to indicate whether the shift was accomplished via a change in the light or dark phase, respectively (Aschoff et al., 1975). For example, a 5-h phase delay made by extending the light phase is indicated by –5L. ZT (zeitgeber time), ZT0 = baseline (BL) time of lights-on. **(B)** Coronal brain sections of mRNA hybridization for *c-fos* and *per1* from hamsters that were entrained (ENT), free ran in the LD cycle (FR), or were circadian-arrhythmic (ARR). **(C)** The percent of animals that reentrained (left panel) and the duration of their active phases (α, right panel) after given a 2-h light pulse given early (E), middle (M), or late (L) in the night, and followed by a -3L shift on the following day. **(D)** Representative actograms with consecutive days double plotted from top to bottom of hamsters that reentrained **(D1)** or free-ran after -5L **(D2,D3)**; arrows indicate day of -5L, gray shading indicates nighttime to visualize phase shift. **(E)** Representative actograms of animals that became arrhythmic after the DPS protocol (**E1–E3**, indicated by arrows). **(F)** Mean mRNA values of clock genes (*per1*, *per2*, *bmal1*, and *cry1*) quantified by RT-PCR from the SCN of ENT (red) and ARR (green) hamsters. Error bars removed for clarity. ZT0 = light onset in 16:8 LD cycle. Figures modified from Ruby et al., 1996, 1998, 2004, Barakat et al. (2004, 2005) and Grone et al. (2011).

I’d like to say that I developed the DPS model through a combination of deep thinking and meticulous deployment of circadian principles, but in truth, it was an accident. An undergraduate who worked in my laboratory prepared animals for a study, but had to abandon the project to attend to a family matter. After a few weeks, I delayed the light-dark (LD) cycle in the animal room by 5 h so that the lights would come on at the more user-friendly time of 0700 h instead 0200 h (Figure 1A). Being preoccupied with other more pressing studies, the project languished. When I plotted the actograms a few weeks later, I found that 10 animals were free-running *through the LD cycle* at periods ranging from 24.3–26.3 h (Ruby et al., 1996). One hamster became completely ARR immediately, while the remaining animal reentrained to the LD cycle (Ruby et al., 1996). If not for that last animal, I would have thrown the data in the trash and started over. But of course, if one animal reentrained, then the lights must be working properly, otherwise, to what zeitgeber was it entrained? Just in case, I went into the lab one morning to watch the lights come on at 7 AM, checked them every hour throughout the day, and waited 16 h to watch them shut off at 11 PM. The lights were fine. Moreover, there were no signs of relative coordination to suggest any photic modulation of the pacemaker.

To get some perspective on these observations, I sent the data to Ben Rusak and Jeff Elliott, to whom I am indebted for their thoughtful insights and encouragement. In addition, Jeff generously shared some of his unpublished data with me that shed light on the hamster entrainment problem. In a past study, Jeff entrained golden hamsters to *T*-cycles of 23 h (0.25 h of light: 22.75 h of dark). Most animals shortened their active phases (i.e., alpha, α) to <4 h (i.e., α compression), at which point entrainment was lost as the animals free-ran through the *T*-cycle. When Jeff subsequently transferred the animals into constant darkness (DD) and administered light pulses across their circadian cycles, he found that they had a flat phase response curve (PRC; Elliott, presented at the International Chronobiology Conference, 1999). Some animals, however, did not exhibit α compression or flat PRCs and thus remained entrained to the *T*-cycle.

In the Siberian hamsters, I had also observed that α compression preceded the free run through the LD cycle which, based on Jeff’s work, suggested that the pacemaker was no longer responding to light (Ruby et al., 1996). To test for this, I decided against generating a PRC in the free-runners because their rhythms were a little too messy, so it would be easy for small phase shifts to go undetected. Instead, we put the animals in DD for 24 h, and then administered a light pulse (30 min) early in their active phase and probed the SCN for *c-fos* and *per1* mRNA induction because light pulses rapidly induce expression of these genes (Barakat et al., 2004). In contrast to the control animals, we found absolutely no mRNA induction in the SCN of the free-runners (Figure 1B). We placed groups of hamsters in several weeks of DD or constant light (LL), but neither condition had any effect on the period or phase of their rhythms (Figures 1D2,D3). We also checked for photic mRNA induction in a group of ARR animals, assuming we would find the same result as with the free-runners, but, as it turned out, the SCN of ARR hamsters was still responsive to light (Figure 1B; Barakat et al., 2005). Given that the SCN of ARR hamsters was sensitive to light, it is puzzling that light did not restore rhythms to these animals. As will be discussed later, it also suggested that the underlying mechanism of arrhythmia is different between LL and DPS-induced arrhythmia.

As most students of circadian rhythms know, light in the middle of the night can suppress the amplitude of the pacemaker (Jewett et al., 1991; Honma and Honma, 1999; Leloup and Goldbeter, 2001; Huang et al., 2006). If the light intensity and timing are just right, the light pulse sends the pacemaker into a state of arrhythmia (i.e., singularity). I house my hamsters in 16 h of light and 8 h of dark, thus, on the first day of a 5-h delay of the LD cycle, light extends through the middle of the night (Figure 1A), so I decided to test the idea that light exposure in the middle of the night could break entrainment (Figure 1A). My results matched Jeff’s; animals that compressed α in response to the 5-h delay of the LD cycle lost entrainment, while animals that maintained an uncompressed α successfully reentrained (Ruby et al., 2004). By contrast to a 5-h delay, all hamsters easily reentrained to a 3-h delay of the LD cycle because, presumably, light did not impinge on the middle of the night (Figure 1A). To test that idea, hamsters were pre-treated with 2-h of light in the middle of the night, and then, on the next day, the LD cycle was delayed by 3 h to see if the light pulse would impair reentrainment (Figure 1A). As controls, I ran two additional groups that were given the 2-h light pulse early or late at night (Figure 1A). As it turned out, I was wrong. The light pulse late at night had the greatest impact on reentrainment—none of those animals reentrained. By contrast, all animals reentrained if they got the light pulse early at night, and 58% of animals reentrained if they got the light pulse in the middle of the night (Figure 1C, left panel). The percent of animals that reentrained also correlated with the extent of their α compression (Figure 1C, right panel). And just as Jeff found, when α was compressed to <4 h as in our “late” group, none of the animals reentrained (Figure 1C). Of those animals, ∼30% free-ran and ∼70% became ARR (see Figures 1D,E for examples).

A similar finding was reported by Steinlechner et al. (2002) using a comparable method, but in their protocol, hamsters were given a 15-min light pulse late at night, and then another light pulse early on the following night. The combination of advancing, then delaying, signals caused a rapid compression of α followed by arrhythmia in body temperature, locomotor activity, and melatonin secretion. Since these studies were published, a similar relationship between α compression and entrainment was found in an experiment on selective breeding in Siberian hamsters. Animals with a significant delay in their time of their nightly activity onset were bred to increase this phenotype in a laboratory population (Weinert and Schöttner, 2007). The most common phenotype exhibited a spontaneous gradual compression of α over a period of several weeks (Weinert and Schöttner, 2007). A number of animals compressed α down to <3 h. At that point, some animals began to free run with periods close to 25 h, whereas other hamsters became ARR.

To summarize, severe α compression precedes both loss of entrainment and arrhythmia. In Siberian hamsters, the key to arrhythmia is about maximizing alpha compression, and the most severe alpha compression results from the combined effects of a phase-advancing and a phase-delaying signal occurring (in that order) within a single circadian cycle. Furthermore, housing free-running or ARR hamsters in DD does not restore entrainment (Figure 1D3; Steinlechner et al., 2002; Barakat et al., 2004). The combination of a 2-h light pulse late at night, followed by a 3-h delay of the LD cycle on the next day, termed the DPS protocol by Prendergast et al. (2012), reliably induces arrhythmia in 40–65% of hamsters in any given cohort.

## Why Arrhythmia? Why This Species?

Steinlechner et al. (2002) explain the progression from alpha compression to arrhythmia in the central pacemaker in the context of the two-oscillator model originally described by Pittendrigh and Daan (1976) and also used by Elliott and Tamarkin (1994) to describe circadian control of wheel-running and melatonin secretion in Syrian hamsters. In nocturnal rodents, α is determined by the phase-angle difference (ψ_EM_) between the evening (E) and morning (M) oscillators which control activity onset and offset, respectively, in a multioscillator pacemaker. The key to the model is the coupling strength between these oscillators. Tight coupling means that an advancing or delaying light signal phase-shifts both E and M in unison, without any changes in ψ_EM_ (Pittendrigh and Daan, 1976). Loose coupling allows for changes in ψ_EM_ and in α because E and M can phase shift independently of one another. Photic signals compress α by delaying E and/or by advancing M, which then decreases ψ_EM_. Steinlechner et al. (2002) suggest that arrhythmia arises when ψ_EM_ is driven to zero. In that condition, the pacemaker can be considered to be in a state of singularity with zero amplitude.

We found evidence for the amplitude suppression hypothesis by quantifying clock gene expression in the SCN of DPS hamsters (Grone et al., 2011). DPS animals lacked daily rhythms in expression of *per1*, *per2*, *bmal1*, and *cry1* mRNA (Figure 1F). Moreover, the mRNA levels of those genes were suppressed to a basal level of expression. This is an important point because if SCN neurons were oscillating, but desynchronized from one another, mRNA levels in DPS hamsters would be expressed well above basal levels. In mice made ARR by LL, neuronal desynchrony in the SCN was reported using *Per1:GFP* fluorescence to monitor individual cells (Ohta et al., 2005). In those animals, individual SCN neurons continued to oscillate at circadian periodicities, but phase synchrony was widely dispersed in the neuronal population, resulting in ARR locomotor activity patterns. Taken together, the underlying mechanisms of arrhythmia caused by LL versus the DPS protocol appear to be quite different. If ARR hamsters truly are an instance of amplitude suppression, then it suggests that the clock in each SCN neuron stopped oscillating, which is an idea that was unheard of not that long ago.

I have been asked many times if the DPS protocol could be adapted for mice. I am doubtful that it can, mainly because most mice have such small phase advance zones, if any at all. Worse, one would need to compress α by putting mice into a photoperiod with nights much shorter than 12 h, which would make the phase advance zone even smaller (i.e., reduce PRC amplitude; cf., Elliott, 1976). And even if one could figure out how to make the advance zone larger, oscillator coupling in the SCN would have to be loosened up, although there might be practical ways to do so (Herzog et al., 2017). I also would not suggest using LL because it is stressful and not reliable. I once housed a group of C57BL/6 mice in LL under 1,000 lux of light and all of them free ran for 6 months without any signs of arrhythmia. Having said all this, producing arrhythmia in a rodent is not impossible (Honma and Honma, 1999), but keeping them ARR for months while housed under a standard LD cycle might be.

For the sake of posterity, I would like to point out that Stephan Steinlechner and myself both discovered the phenomenon of using advancing and delaying light signals to induce long-term arrhythmia independently and without knowledge of the other’s work. At a meeting of the Society for Research on Biological Rhythms (1998) where I presented some of these research findings, Franziska Wollnik informed me that Stephan Steinlechner had already been making Djungarian hamsters ARR for years, which was news to me. I was never able to confirm this with him, but I assume that Franziska was right.

It is difficult to explain all of these data according to current theories of entrainment, and harder yet to provide a unifying physiological mechanism that ties all the data together, but there is a related question we should consider. If circadian rhythms and their entrainment to light are so adaptive, then why is entrainment, and the pacemaker itself, so vulnerable to light, particularly in a seasonal species that depends on proper entrainment to changing daylengths for its survival? I suspect that what we are seeing in the laboratory is a window into some adaptation, one that promotes survival in animals from harsh environments. During the breeding season, nights become very short at the high northern latitudes from where Siberian hamsters originate. Females of this species experience high energetic demands as they can be pregnant and nursing a litter of pups simultaneously. Short nights limit foraging time, so in periods of food scarcity, it might be beneficial to forgo strict nocturnality and risk predation to forage during the day. In this situation, a labile circadian pacemaker would be adaptive to permit daytime activity. In Siberian hamsters, reducing food intake weakens circadian timing in body temperature and activity as the animals redistribute their activity into the daylight hours (Ruby and Zucker, 1992; Challet et al., 2000). By contrast, the same reduction in food intake in Syrian (golden) hamsters does not (Challet et al., 2000). Rather, that species, which originates from a more temperate climate, maintains strict nocturnality, but compensates for food shortages by reducing daily amounts of locomotor activity. This speculation raises a worthwhile issue: if a weakened circadian system is needed to permit foraging into the daytime hours, then impairments in circadian timing should have no effect on spatial memory or on hippocampal function which are required to form a cognitive map of food sources. But, as we shall see, the relationship between circadian rhythms and the hippocampus is not so simple.

## SCN Lesions Improve and Impair Memory, or Neither

Research on circadian rhythms and memory goes back nearly 50 years, all of which has been well summarized in a pair of excellent reviews (Smarr et al., 2014; Krishnan and Lyons, 2015), so there is no need to summarize that work here. As those reviews show, the majority of work performed with rodents employed memory tasks that critically depend on the hippocampus. In general, those studies found that task performance varies across the day and night, and perturbations to circadian timing, like simulated chronic jet-lag, impair task performance. These findings led to the idea that the circadian system exerts some degree of control over information processing in the hippocampus. To directly test this hypothesis, several studies have evaluated memory performance in animals in which the SCN had been surgically ablated (SCNx). But as I will argue, the bulk of the data show that SCN ablation does not impair memory. In fact, some studies show that SCN ablation *improved* memory.

**FIGURE 2:**
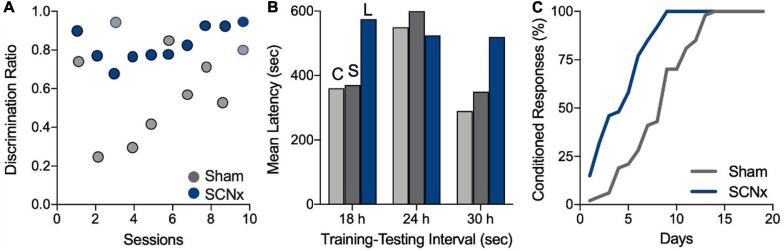
SCN lesions appear to improve memory. **(A)** Rats were trained in a discrimination task that required them to obtain a food reward from one feeder in the morning and another feeder in the afternoon. Higher discrimination scores indicate better memory retention. Circles represent mean scores for each group by session. SCNx rats (blue) appeared to acquire the discrimination in fewer sessions than did sham-operated rats (gray). Figure modified from Mistlberger et al. (1996). **(B)** Rats in a passive-avoidance task learned to avoid entering a dark box where they previously received a foot shock. Longer latencies to reenter the dark box signify better memory of the shock. SCN lesions resulted in longer latencies and appeared to improve memory of the shock at 18 or 30 h after training (C, control; S, sham-lesioned; and L, SCN-lesioned). Figure modified from Stephan and Kovacevic (1978). **(C)** Rats were trained to replace a conditioned reflex to sound with one conditioned to a fixed time interval of 20 s. Training sessions consisted of 20 trials/day over 1 month. The authors statistical analysis showed that SCNx rats acquired the response significantly faster than sham-operated animals. Figure modified from Vodolazhskaya et al. (2001). All figures redrawn from published data and are for illustration purposes only.

A summary of memory tasks performed with SCNx animals reveals some trends across studies (Table 1). First, in 11 of 14 memory tests from 11 publications, the authors reported no memory impairments in SCNx animals. Second, three of these studies can easily be interpreted to show that SCN ablation improved memory (Figure 2). In the study by Mistlberger et al. (1996), rats were trained to use time of day as the discrimination cue for obtaining food by pressing a lever from two food dispensers, one in the morning and one in the afternoon. The data over the first ten training sessions shows that SCNx animals acquired the time-of-day memory faster than SCN-intact animals (Figure 2A). In a passive-avoidance test by Stephan and Kovacevic (1978), memory recall was optimal 24 h after training, but poor at other non-circadian intervals. SCN lesions eliminated this time-of-day effect by improving performance at those non-circadian times (Figure 2B). In a time-memory task where rats were trained to respond to a sound after a fixed time interval, SCNx animals acquired the conditioned reflex significantly faster than SCN-intact animals (Figure 2C; Vodolazhskaya et al., 2001). Taken at face value, these three studies show that SCN ablation improved memory, thus contradicting the expectation that SCN lesions should impair memory processing.

**TABLE 1:** Memory tests performed in SCNx animals.

**Memory task**	**Model**	**Recovery time (days)**	**Reported memory Impairment?**	**References**
NOR	B6/C3H mouse	>21	No	Chuluun et al., 2020
NOR	Siberian hamster	>28	No	Fernandez et al., 2014
SA	Siberian hamster	>28	No	Fernandez et al., 2014
PA	Golden hamster	>21	No	Cain et al., 2012
CPA	Golden hamster	>21	No	Cain and Ralph, 2009
MWM	C57BL/6 mouse	14	No	Phan et al., 2011
cTPL	C57BL6/J mouse	>17	No	Mulder et al., 2014
TMh	Wistar rat	>21	No	Maruyama et al., 2007
PA	Sprague-Dawley rat	7–9	No	Stephan and Kovacevic, 1978
TDF	Wistar rat	?	No	Mistlberger et al., 1996
CR	Mongrel white rat	?	No	Vodolazhskaya et al., 2001
NOR	C57BL/6 mouse	14	Yes*	Phan et al., 2011
CFC	C57BL/6 mouse	14	Yes*	Phan et al., 2011
NOR	C57BL/6 mouse	>14	Yes*	Shimizu et al., 2016

TDF, time of day discrimination for food reward; PA, passive avoidance; NOR, novel object recognition with 24 h training-testing interval; SA, spontaneous alternation; CPA, conditioned place avoidance; CR, conditioned reflex; TMh, time memory to heat exposure; MWM, Morris water maze; cTPL, circadian time place memory; CFC, contextual fear conditioning; and RT, recovery time allowed after SCN lesion surgery (days). *non-cognitive or methodological factors qualify the result (see text for details). ?, information unavailable. SCNx studies of food entrainment and of individual human medical cases were excluded.

If SCN lesions can improve memory, can they also impair memory? Memory impairments were reported for SCNx mice by Phan et al. (2011), but closer evaluation allows a different interpretation. In their test of novel object recognition (NOR), mice were allowed to explore two identical objects for 5 min on each of two consecutive days. On the third day, one object was replaced with a new unfamiliar object. In a typical NOR task, control animals will spend about twice as much time exploring the new object compared to the familiar one, indicating a memory for the familiar object. In this study, SCNx mice spent the same amount of time with the old familiar object as they did with the novel one, indicating a failure to either encode or recall the “familiar” objects. However, the control (sham-operated) animals also failed to recognize the familiar object 24 h after training. This might be due to the brief amount of time allowed for exploration (5 min). Most NOR studies with mice allow 10–15 min for object exploration because their latency to engage the objects can be several minutes, even with habituation to the arena. As a result, studies which allow at least 10 min of object exploration time routinely retain object memory for 24 h (c.f., Fernandez et al., 2007; Antunes and Biala, 2012; Lueptow, 2017; Chuluun et al., 2020). By contrast, Siberian hamsters perform successfully in the NOR task with only 5 min of exploration time because they tend to engage objects almost immediately upon entry into the arena (Ruby et al., 2008).

In a test of contextual fear conditioning (Phan et al., 2011), SCNx mice froze significantly less and were more active than controls in the test session, suggesting impairment of context association for the footshock. It is possible, however, that the decrease in freezing was due to the relatively short postsurgical recovery time of 14 days, which is shorter than similar studies (Table 1). Alternatively, few chronobiologists know that SCN lesions make some animals hyperactive, which would explain the decrease in freezing. We screen our SCNx and sham-operated animals for hyperactivity by checking for postsurgical increases in home cage locomotor activity. More puzzling though, is that SCNx mice performed just as well as controls in the Morris water maze (MWM), showing no differences in escape latencies, which is the gold standard for evaluating hippocampal-guided navigation in the MWM. Thus, the normal escape latency shows that navigational functions in the hippocampus and exploration behavior of SCNx animals were intact.

There is another intriguing facet to the Phan et al. (2011) study. The authors suggest that the loss of circadian rhythms in mitogen-activated protein kinase (MAPK) in the hippocampus is a cause of the putative memory impairments. But there is another way to interpret the data. The unimpaired performance of SCNx mice in the MWM indicates the persistence of robust spatial memory and hence, intact hippocampal processing. Taken together with the inconclusive evidence from the NOR and fear conditioning tasks, one could just as easily conclude that circadian rhythms in the hippocampus are unnecessary for information processing in that structure. Furthermore, if we assume that SCN lesions also eliminated MAPK rhythms in all the other memory studies of SCNx animals (Table 1), then the lack of memory impairments in the other 11 memory tests allow us to conclude that that the hippocampus does not require circadian rhythms in MAPK for successful performance in those memory tasks. MAPK is an important component of memory storage, but based on all of the SCN lesions studies (Table 1), daily cycles in its expression do not appear necessary for the hippocampus to function normally.

Results from the remaining SCNx studies in Table 1 are at best contradictory. The study by Shimizu et al. (2016) is the outlier. The authors reported that SCNx mice recalled a familiar object after 8 min, but not after 24 h. The actual exploration times are not given, so it is possible that the SCNx mice were less interested in the objects than were the control animals, or that they had longer latencies to explore the objects. This study set a minimum time of 10 s total for exploration of both objects (i.e., total duration of physical interaction with the objects), whereas a 20 s minimum is recommended (Lueptow, 2017). Moreover, trisomic (Down Syndrome) Ts65Dn mice exhibit memory deficits in object recognition (Fernandez et al., 2007), but when trained and tested weeks after SCN ablation and given 10 min of exploration time, they were able to recall the memory of an object 24 h after exploring it (Chuluun et al., 2020). More to the point here, SCN ablation did not impair 24-h recall in the control group of 2N diploid littermates (Chuluun et al., 2020).

Attributing memory deficits to SCN ablation requires ruling out all non-cognitive explanations, but this cuts both ways—the aforementioned memory improvements in SCNx animals require the same scrutiny. Did SCN lesions really improve memory in those three studies? Cain and Ralph (2009) performed a replication of the study by Stephan and Kovacevic (1978) in which it appeared that SCN lesions improved memory, but they were unable to replicate that finding. To explain this discrepancy, Cain and Ralph (2009) suggest that the high performance (i.e., long step through latencies) of SCNx animals in the study by Stephan and Kovacevic (1978) was not due to improved memory, but was an artifact of the size and placement of the lesions which may have damaged the optic tracts. Such damage could impair light perception, thereby reducing the aversiveness of the brightly illuminated start box and hence, increasing the latency to escape. In regards to the study by Mistlberger et al. (1996), the putative memory improvement in SCNx animals might be explained by the fact that SCNx animals underwent more training sessions than did the SCN-intact controls (Mistlberger, personal communication). This piece of information was not reported because the rate of discrimination acquisition was not relevant to the goals of that study. Vodolazhskaya et al. (2001) appears to be the only study to show a bona fide improvement in memory performance in SCNx animals. However, we do not know if time of day might have affected acquisition of the conditioned response in the control animals, and, therefore, whether the sham-operated animals would have performed better had they been trained and tested at other times of day.

Methodological differences among these studies that could explain the seemingly contradictory results such as lesion size and placement, postsurgical recovery time, amount of training, latency to engage the task, and time of day that tests were done, etc., still might not resolve these issues. Discrepant findings in behavioral studies have plagued many areas of neuroscience, despite extraordinary efforts to standardize conditions across laboratories (Crabbe et al., 1999). The standardization of behavioral tests has been suggested to minimize this problem—and that approach has its advantages, but it is doubtful that differences among laboratories can be minimized to the point where they are no longer problematic (Wahlsten, 2001). Although discrepancies exist, there is far more consistency across laboratories in primary findings (Crabbe et al., 1999). Thus, the interpretation of individual studies of SCN lesions and memory, no matter how comprehensive they appear, must still be evaluated within the larger context of all other relevant SCN lesion studies in order to minimize differences in testing procedures and conditions that vary across laboratories. In that context, it is clear that the overwhelming weight of evidence shows that SCN lesions do not impair memory processing in hippocampal-dependent tasks (Table 1).

## A Malfunctioning SCN Is Worse Than No SCN

If SCN lesions do not impair memory then why have so many studies reported that chronic disruption of circadian timing impairs memory? Further, why is there mounting clinical evidence that circadian dysfunction contributes to cognitive decline in aging (Tranah et al., 2011; Covell et al., 2012; Coogan et al., 2013)? I propose that the answer to these questions is that an intact, but malfunctioning, SCN interferes with cholinergic transmission in the septohippocampal pathway. In this section, I will discuss how the DPS model has shed light on the functional relationship between the SCN and hippocampus, and describe a mechanism to explain how a dysfunctional SCN interferes with memory.

We evaluated memory in DPS hamsters with the NOR task and by spontaneous alternation (SA), which assays spatial working memory. In the NOR task, we allowed the hamsters 5 min to sample two identical objects (sample phase), and then returned them to the arena after a fixed time interval (i.e., 1, 20, or 60 min, or 24 h), and allowed them to explore two objects for 5 min (test phase); one object was from the sample phase (i.e., familiar) and the other was different (i.e., novel). Entrained hamsters spent an equal amount of time with both objects in the sample phase, but spent nearly twice as much time with the new object, thus indicating memory retention of the familiar object (Ruby et al., 2008).

The SA task is less well known than the NOR task, but is better suited as a behavioral assay of hippocampal function, as will be discussed later. We used a continuous trials version of the SA test in which animals are placed in a start box at the base of a T-maze for 1 min, and then allowed to explore the maze for 7 min. Alternation behavior is the tendency of animals to alternate left-right arm choices with each trip from the start box to the maze intersection at the top of the “T.” An alternation event is when an animal chooses, for example, the left arm on its first trip through the maze, then the right arm on its next trip. Performance is scored as the proportion of trips from the stem to the intersection where an animal alternates arm choices, compared to when it chooses the same arm (i.e., non-alternation).

**FIGURE 3:**
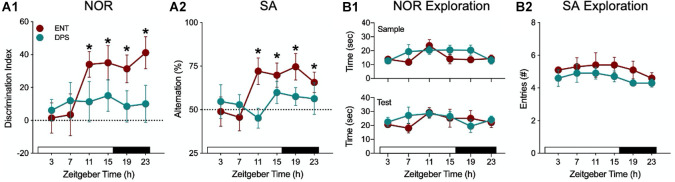
Recognition and spatial working memory were impaired in DPS hamsters. **(A)** Performance of ENT hamsters (red) and DPS (green) hamsters in the NOR **(A1)** and SA **(A2)** tasks at different times of day. * indicates that task performance was significantly different from chance (*P* < 0.05; dotted line). In contrast to task performance, exploration behavior, defined as the amount of time spent exploring objects **(B1)** and number of arm entries **(B2)**, did not change across the day and did not differ among ENT and DPS hamsters. NOR task used a 60 min interval between sample and test phases. Figure modified from Ruby et al. (2008, 2013).

Both memory tasks showed that there is a daily rhythm in performance in ENT hamsters, which was poor early in the day, but successful at the remaining time points (Figures 3A1,B1). DPS hamsters, however, failed at all time points. By contrast, exploratory behavior, given as the time spent exploring objects (NOR) or as the number of arm entries in the maze (SA), was constant across all time points (Figures 3A2,B2). Moreover, the amount of exploration was the same among ENT and DPS hamsters. Both tasks reveal two important features of circadian involvement in memory tests of exploration. First, while test performance varies across the day, exploration behavior does not; it remains constant across the day and night. Second, loss of circadian timing did not impair the motivation to explore the objects and environments. DPS hamsters fail, but not for lack of trying; they explore just as much as ENT hamsters.

**FIGURE 4:**
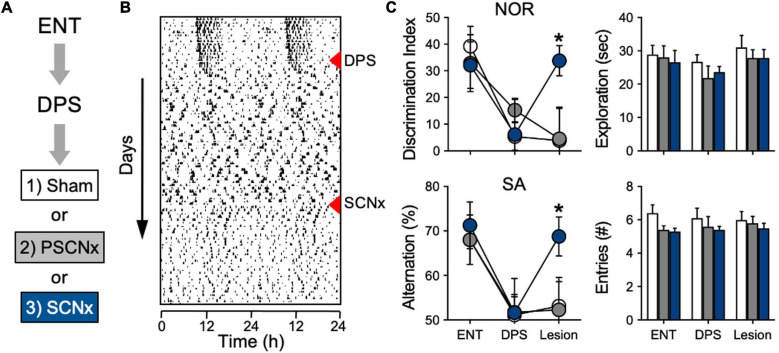
SCN lesions rescue object recognition and spatial memory in DPS hamsters. **(A)** Sequence of experiment stages. ENT hamsters performed the NOR and SA tasks, followed by exposure to the DPS protocol (red triangle). 4 weeks later, arrhythmic animals underwent SCN ablation or Sham surgeries (red triangle). **(B)** Representative actogram of an SCNx animal; memory tasks were performed at the end of each experimental stage. NOR task used a 24-h interval between sample and test phases. **(C)** Memory was rescued only in animals with complete SCN ablation (blue circles). No differences were found in NOR exploration times or in SA arm entries; all tests were performed within 3 h before dark onset. Figure modified from Fernandez et al. (2014).

Given the observed differences in SCNx animals and DPS hamsters in memory performance, we decided that a head-to-head test of both types of arrhythmia in the same species, in the same laboratory, and under identical conditions might clarify the role of the SCN in the NOR and SA tasks. As expected, we found that performance in the NOR and SA tasks was fully intact in SCNx animals, but impaired in DPS hamsters (Fernandez et al., 2014). There were also no differences between these groups in their behavior during the tasks; both groups spent the same amount of time exploring objects and the maze (Fernandez et al., 2014). Those results suggested that memory impairments in DPS animals were caused by arrhythmia in the SCN. We tested this hypothesis by evaluating whether ablation of the SCN could rescue memory in DPS animals (Figure 4A). A group of hamsters was first tested while they were entrained (ENT), after which, they were exposed to the DPS protocol. Hamsters that were completely ARR 4 weeks later were tested a second time. In the third phase, those DPS hamsters underwent SCN lesion surgery. After 4 weeks of recovery from surgery, this group of hamsters were tested a third time on both memory tasks (Figure 4B). Complete SCN ablation rescued memory in DPS hamsters. Hamsters that sustained complete bilateral ablation of the SCN performed as well as sham-operated animals in both tasks (Figure 4C). There were no significant changes in exploration behavior across the three phases of the experiment (Figure 4C).

Among the lesioned hamsters were a subgroup of animals that sustained only partial damage to the SCN (20–60% ablated). In that group with partial SCN lesions (PSCNx), damage to the SCN did not rescue memory (Figure 4C). This finding was not entirely unexpected. It is well documented that partial ablation of the SCN does not eliminate circadian timing, with some studies showing that only 10% of the SCN is sufficient for circadian rhythms to persist (cf., Ruby, 2003). The PSCNx animals showed that—just as with circadian rhythm generation—a small subset of SCN neurons are sufficient to maintain function. But in this case, that function is memory impairment. Taken together, these experiments show that SCN lesions do not impair long-term object recognition or spatial working memory. Moreover, they also show that an ARR SCN can interfere with memory processing even when most of the nucleus is destroyed.

## Spontaneous Alternation: A Great Memory Task for Chronobiologists

**FIGURE 5:**
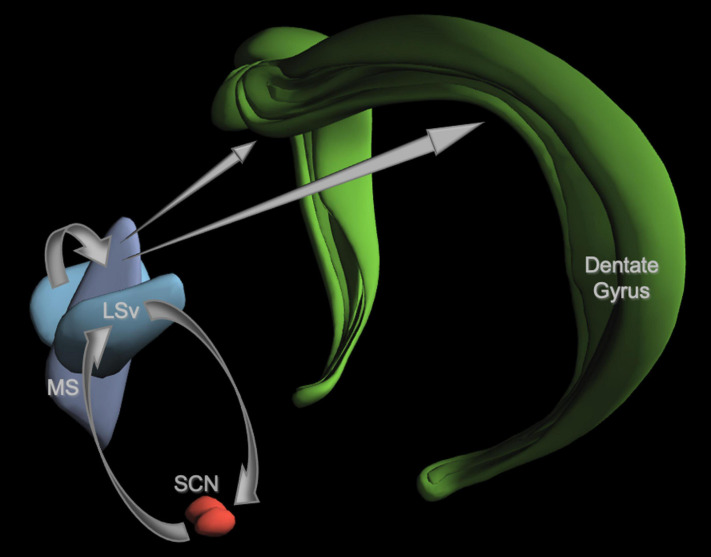
A neuroanatomical model of a proposed SCN-septum-dentate circuit. For the sake of clarity, only the elements of the model discussed in the text are illustrated here. The projections from each structure are shown as gray arrows. The SCN has reciprocal connections with the ventral lateral septum (LSv). GABAergic fibers from the LSv innervate the medial septum (MS) shown here without the diagonal band of Broca. MS fibers containing Ach, GABA, and glutamate project to all subregions of the hippocampus, but only the dentate gyrus is shown. Fibers from the LSv to the SCN are primarily vasopressinergic (VP). See text for details and citations. Image credit: Allen Institute.

Spontaneous alternation behavior is a memory task well-suited to circadian studies, so it is worth taking a moment to provide some background. SA behavior is mainly used as an assay of spatial working memory. As such, it is an excellent metric for the immediate and long-term effects of a treatment, such as jet-lag, on memory. SA tasks allow for frequent (daily) re-testing of individual animals without decrements in performance (Douglas, 1989). Unlike fear conditioning or food rewards, SA does not produce arousal or require training sessions, all of which can produce daily timing signals. It is also highly sensitive to disturbances in cholinergic pathways from the medial septum (MS) to the dentate gyrus (DG) of the hippocampus. The only known projection of the SCN to the entire limbic system is not to the hippocampus, but to the ventrolateral subregion of the lateral septum (LSv) which has reciprocal projections to the SCN (Watts et al., 1987; Watts, 1991; Morin et al., 1994; Kriegsfeld et al., 2004; Figure 5). GABAergic fibers from the LSv target the MS (Risold and Swanson, 1997), which is a major source of acetylcholine (Ach; Dutar et al., 1995; Teles-Grilo Ruivo and Mellor, 2013). Cholinergic projections from the MS innervate the DG and CA regions of the hippocampus (Haam and Yakel, 2017), but it is the projections to the dentate that underlie SA behavior (Gold, 2003; Solari and Hangya, 2018). Because this circuit is essential for SA behavior, the SA task is essentially a biometric readout of the integrity of this circuit.

I am indebted to my colleague, Fabian-Xosé Fernandez, for introducing me to the literature on SA behavior and for his collaborative efforts in developing our working model describing the functional connectivity between the SCN and hippocampus (Ruby et al., 2008; Fernandez et al., 2014). A more detailed primer on SA behavior compiled by Dr. Fernandez may be found in Ruby et al. (2017, Supplementary Material). Briefly, SA behavior was first observed in rats over 100 years ago (Hunter, 1914). Since that time, it has been reported for insects, crustaceans, fish, reptiles, birds, rodents, and humans (Hughes, 1989; Lewis et al., 2017). Animals are intrinsically motivated to keep track of information relating to the location of food, predators, and potential mates (Barnett, 1958; Berlyne, 1966). These behaviors, collectively termed *exploration*, ultimately result in the creation of a cognitive map, which is a complex internal representation of the environment’s physical space (Tolman, 1948; O’Keefe and Nadel, 1978, 1979). Animals continue to update their spatial representations of places over time, maintaining the accuracy of these representations via regular bouts of patrolling (Nadel, 1990). If the environment they are patrolling is given a specific spatial framework, such as a T-maze, they will travel systematically from one endpoint of the apparatus to the next, alternating more than two-thirds of the time (Tolman, 1925; Gerlai, 1998).

A synthesis of work done over the past five decades suggests that cholinergic input from the MS to the DG is critical for maintaining this function. The developmental emergence of SA behavior and of hippocampal maturation occur in parallel in several species (Douglas et al., 1973; Bronstein et al., 1974; Frederickson and Frederickson, 1979; Dumas, 2004; Blair et al., 2013), *including humans* (Vecera et al., 1991). The latest developing subfield of the hippocampus is the DG; the granule cells there (*fascia dentata*) still divide after birth in many species to sculpt a portal of information flow from the medial entorhinal cortex (MEC)—another component of the brain’s navigation system—to the hippocampus. In the rat, SA rates increase as the dentate matures (Douglas et al., 1973). The achievement of adult-like SA performance at the end of the third postnatal week coincides with increases in the synaptic strength of the MEC-DG perforant path (Dumas, 2004).

Rodents with electrolytic or excitotoxic lesions of the MS do not alternate significantly above a chance rate of 50% (Douglas and Raphelson, 1966; Clody and Carlton, 1969; Dalland, 1970; Johnson et al., 1977; Thomas, 1979; Brito and Thomas, 1981; Hepler et al., 1985; Chang and Gold, 2004). Animals treated systemically with anticholinergic drugs, such as scopolamine, also perform poorly in a T or Y-maze task (Squire, 1969; Leaton and Utell, 1970; Douglas and Truncer, 1976; Kokkinidis and Anisman, 1976; McNaughton and Feldon, 1980; for a comprehensive review, see Klinkenberg and Blokland, 2010). These data—collated from adults—complement studies from developing rat pups, which show that SA behavior comes online along the same time-course with which the maturing hippocampus is innervated by the septum (Kirkby, 1967; Egger et al., 1973; Hess and Blozovski, 1987). Research by the Gold lab suggests that the septohippocampal pathway is highly active during SA and is taxed more when SA behavior is tested in mazes with more arms. Rats exhibit significant spikes in hippocampal Ach release during SA in a four-arm plus maze that is sustained throughout the testing period (a 50–70% increase relative to baseline as determined by *in vivo* microdialysis; Ragozzino et al., 1994, 1996, 1998; Ragozzino and Gold, 1995; Stefani and Gold, 2001).

In summary, the septohippocampal pathway—particularly its cholinergic projections—is considered to be the critical structure for expression of alternation behavior because: (1) there is a close association between maturation of septal inputs to the DG, and development of the dentate itself, that is necessary for SA behavior, (2) only brain lesions that destroy or alter the circuits associated with this pathway impair SA behavior, whereas damage to several other brain regions outside of the hippocampal system do not, and (3) pharmacological inhibition of septal inputs to the dentate impair SA behavior.

## The Importance of Being Non-Circadian: Theta Oscillations

The deficits in the SA task observed in DPS hamsters implied that there should be functional deficits in cholinergic septohippocampal circuitry as manifested in the expression of theta rhythms. Theta oscillations in this circuit coordinate the firing patterns of neuronal ensembles across diverse brain regions. This synchronized activity allows information stored in anatomically distant regions to be shared and processed as coherent memories (Colgin, 2016). Theta rhythms dominate the electroencephalogram (EEG) during activities that support working memory and the encoding of episodic memories, such as when animals explore novel objects or navigate their environment. Ach signaling supports spatial working memory (Ragozzino et al., 1998; Gold, 2003; Teles-Grilo Ruivo and Mellor, 2013) via its release from medial septal neurons onto cells in the DG and CA1 (Dutar et al., 1995; Teles-Grilo Ruivo and Mellor, 2013). This phasic release of Ach from septal neurons is highly correlated with theta frequency (Lee et al., 2005; Zhang et al., 2010). Furthermore, optogenetic activation of septal cholinergic neurons enhances theta oscillations in hippocampal neurons (Vandercasteele et al., 2014). Thus, we examined whether the loss of circadian timing disrupted theta episodes during object exploration.

**FIGURE 6:**
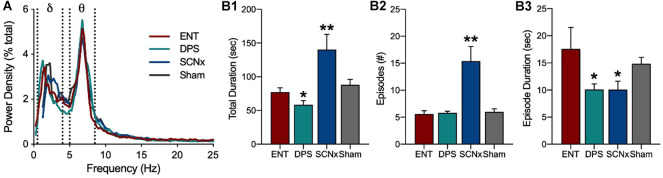
Loss of circadian timing fragments theta oscillations. **(A)** Power density in the EEG averaged over 5 min of object exploration. Delta (δ) and theta (θ) power bands defined by dotted lines. Data for ENT, DPS, SCNx, and Sham groups are aligned by peaks in theta power. Theta dominance was defined by a theta/delta (T/D) ratio > 1.0 within each EEG epoch. **(B)** Theta dominance in the EEG during exploration of a novel object given for **(B1)** total amount of time T/D > 1.0, **(B2)** number of theta-dominated episodes, and **(B3)** the mean duration of individual episodes where T/D was > 1.0. Data are mean (± SEM). * indicates *P* < 0.05, **P < 0.01 compared to ENT. Figure modified from Loewke et al. (2020).

Activity in the EEG delta band (0.5–4.0 Hz) reflects a restful state whereas theta (5–8 Hz) reflects a state of attention and active information processing. Stimuli that increase theta also suppress adjacent frequency bands, thus, the theta/delta (T/D) ratio provides an index of active engagement with environmental stimuli. A single theta episode was defined as the number of consecutive EEG epochs (4 s) in which the T/D ratio was >1.0 such that, for example, two consecutive epochs would be a single theta episode of 8 s. We found that the relative power densities in the EEG spectra did not differ among ENT, DPS, SCNx, or Sham-lesioned animals (Figure 6A), indicating that theta-generating mechanisms were intact in all four groups. The mean duration of individual theta episodes in both DPS and SCNx groups were, however, shortened to half the duration observed in ENT animals (Figure 6B3; Loewke et al., 2020), but DPS and SCNx animals differed in two important respects. First, the total amount of time that theta dominated the EEG during exploration in DPS hamsters was less than it was for ENT animals, whereas SCNx hamsters spent twice as much time in theta (Figure 6B1). Second, the number of theta episodes was the same for ENT and DPS hamsters, but was 3-fold greater for SCNx animals (Figure 6B2).

This shortening, or fragmenting, of theta episodes was a consequence of circadian arrhythmia for both DPS and SCNx groups. Such fragmentation suggests that circadian arrhythmia disrupted signaling in the septohippocampal pathway, possibly by also fragmenting cholinergic signaling in that pathway (Loewke et al., 2020), or by disrupting synchronized firing among neuronal populations (Buzsáki and Moser, 2013; Harris and Gordon, 2015), or perhaps theta fragmentation represents fragmentation in the chunks of information carried within a theta sequence (Gupta et al., 2012; Teng et al., 2018). Deficits in any of these mechanisms of information processing would result in the misrepresentation of spatial information encoded in memory and, perhaps, in the erroneous recall of spatial representations.

The key difference between DPS and SCNx hamsters is that SCN lesions allow the animals to compensate for theta fragmentation by increasing the number of theta episodes through increased cognitive effort, an effect which can increase Ach release from the MS (Ragozzino et al., 1998; Gold, 2003; Roland et al., 2014). The increased frequency of these episodes might support memory by shortening the intervals between theta episodes thus allowing greater continuity in encoding object information. Alternatively, more frequent theta episodes might increase the repetition of information carried in fragmented theta sequences. Regardless of the underlying mechanism, the data show that disruptions to SCN functioning interfere with the normal expression of theta oscillations during active exploration of unfamiliar objects.

## The Arrhythmic SCN: Shifting the Balance in the Hippocampus

The model we developed to explain the memory impairments in the DPS hamsters predicts that these animals should exhibit impairments in cholinergic signaling in the DG of the hippocampus. This assertion is based on data from the theta study discussed above, and on the well-established role of cholinergic projections from the MS/diagonal band to the granule cells in the dentate, which are critical for hippocampal theta, SA behavior, and for spatial working memory (Dember and Richman, 1989; Kesner, 2007; Khakpai et al., 2013; Haam and Yakel, 2017; Sasaki et al., 2018).

**FIGURE 7:**
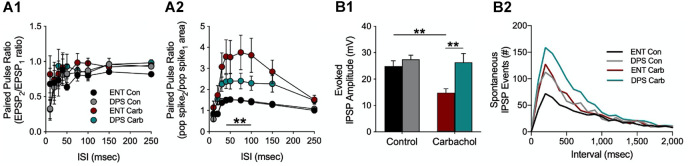
Increased synaptic inhibition and reduced cholinergic responsiveness in the dentate gyrus of DPS hamsters. **(A)** Paired pulse ratios from granule cells in the dentate gyrus at different interstimulus intervals (ISI) during simultaneous recordings of **(A1)** field excitatory postsynaptic potentials (fEPSPs) and **(A2)** populations spikes (POP) under control (Con) or carbachol bath (Carb, 10 μM) conditions. Note that we use area rather than amplitude to quantify the POP spike. This is because amplitude can be confounded both by the number of cells excited by the stimulus and by the synchrony of those cells whereas POP spike area is not. The carbachol-induced population spike increase was attenuated in cells from DPS hamsters compared to cells from ENT animals at ISIs from 40–100 Hz (**P < 0.01). **(B1)** Carbachol normally suppresses evoked inhibition as it did here in the control group (ENT, red); however carbachol had no effect on cells from DPS hamsters (green) where evoked inhibition remained high (**P < 0.001). **(B2)** The number of spontaneous IPSP events in cells from DPS hamsters was 2x that observed in cells from ENT animals under control conditions at intervals < 500 ms (KS test, *P* = 0.014). Carbachol increased spontaneous IPSPs by the same magnitude in both groups. Figure modified from McMartin et al. (2021).

Contrary to my expectations, we found no differences between DPS and ENT controls in long-term potentiation (LTP) in the DG (or in CA1) despite recording from a relatively large number of animals (*n* = 23 DPS, *n* = 24 ENT; data not shown; McMartin et al., 2021). Likewise, there were no differences in excitatory postsynaptic field potentials (fEPSPs) in the dendritic field of the dentate (Figure 7A1), or in population spikes (POP) in the dentate granule (somatic) layer (Figure 7A2), or even in fEPSPs during bath application of the muscarinic cholinergic agonist carbachol (Figure 7A1). However, we began to see differences between the ENT and DPS groups when we examined the effects of carbachol on the POP spike. Carbachol enhanced facilitation of the spike, but that effect was attenuated in the DPS group (Figure 7B). That finding was of interest to us because the postsynaptic signal travels from the dendritic field to the granule cell bodies, and the magnitude of the POP spike generally covaries with the fEPSP that drives them, but that did not happen here. Instead, carbachol had no effect on the fEPSP, but facilitated the POP spike. This suggested that the effects of carbachol on the POP spike must be downstream from the postsynaptic dendritic fields. The most likely explanation for this was that carbachol reduced inhibitory inputs to the granule cell bodies where the POP spike occurs.

This hypothesis was confirmed by direct measures of inhibitory influences on dentate granule cells. Under control conditions, there were no differences among ENT and DPS groups in the amplitude of evoked inhibitory postsynaptic potentials (IPSPs), which is a measure of evoked inhibition (Figure 7B1). Carbachol suppressed IPSP amplitudes in cells from ENT animals, as expected from its known actions on muscarinic receptors (Bell et al., 2013; McQuiston, 2014), but had no effect on cells from DPS animals (Figure 7B1). Furthermore, spontaneous IPSP events, which are a measure of tonic inhibition, were twice as frequent in cells from DPS animals compared to the ENT group (Figure 7B2, for intervals <500 ms). Carbachol increased the frequency of these spontaneous IPSP events, but the magnitude of this increase did not differ among ENT and DPS groups (Figure 7B2). Thus, arrhythmia in the SCN resulted in more than a doubling of the amount of inhibition occurring in dentate circuits, and substantially attenuated the ability of cholinergic agonism to suppress inhibition. This shift toward inhibition would have downstream effects. Dentate granule cell mossy fibers project exclusively to hippocampal area CA3, a microcircuit that is important for spatial memory (Prince et al., 2016; Sasaki et al., 2018; Senzai, 2019). Thus, in DPS animals, there would likely be reduced excitation in that microcircuit, the net result of which would be to impair spatial memory (Flasbeck et al., 2018; Senzai, 2019), which is what we observed in DPS hamsters (Figure 3).

**FIGURE 8:**
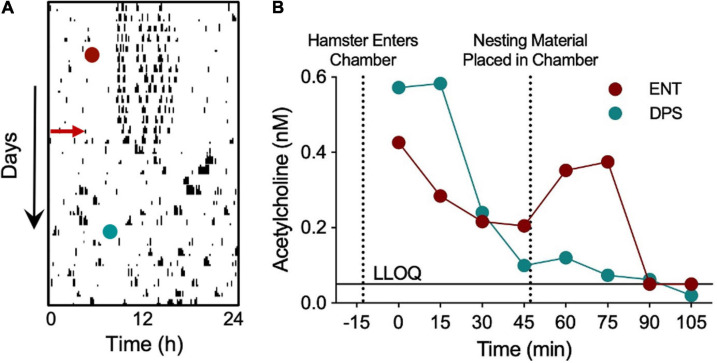
Nest building failed to elicit increases in dentate Ach in a DPS hamster. *In vivo* microdialysis was used to quantify Ach concentrations during nest building in a single hamster before and after the DPS protocol. A microdialysis probe inserted into the dentate gyrus withdrew cerebrospinal fluid (CSF) samples at 15 min intervals, beginning 45 min before nesting material was provided, and continuing for 60 min afterward. Neostigmine (200 nM) was added to the samples to prevent enzymatic degradation of Ach. **(A)** Single-plotted actogram of a hamster before and after the DPS protocol (red arrow). Days and times of microdialysis given (red and green circles). **(B)** Ach concentrations (nM). Dotted lines indicate time when the hamster was placed in the microdialysis chamber and when nesting material was provided. LLOQ, lower level of quantification. Samples quantified courtesy of Brains On-Line (San Francisco, CA, United States).

We further considered whether the increased synaptic inhibition in DPS hamsters might be associated with decreases in Ach signaling from the MS to the dentate so, as part of a pilot study, we performed an *in vivo* microdialysis study to measure Ach in the DG while a hamster was given cotton nesting material in the microdialysis chamber. The nest material serves as a novel object but also requires sustained attention to construct a nest. Siberian hamsters respond almost immediately by separating and fluffing the cotton to construct a nest. Lesions of the septum and hippocampus, among other brain areas, impair nest building (Deacon et al., 2002), thus, we used nesting to promote neural activity in the MS while we collected Ach samples from the DG (see Figure 8A for timeline). Microdialysis samples collected from a single hamster before and after the DPS protocol showed elevations in Ach as soon as the animal was placed in the chamber, then declined over the next 45 min (Figure 8B), at which point, nesting material was provided. Nesting elevated Ach concentrations in the ENT, but not DPS, condition (Figure 8B). Direct observation of the animal did not reveal any obvious differences in its locomotor activity or engagement of the nesting material in the different conditions. As this was only a pilot study, one must interpret the data with caution, but the lack of Ach release suggests that SCN arrhythmia was involved in Ach suppression, despite behavioral engagement with the nest building task.

## Putting the Pieces Together

The collective data from the EEG, hippocampus, and microdialysis experiments suggest a model to explain the memory deficits in DPS hamsters. The encoding of spatial information begins when exploration triggers theta rhythms in the septal nuclei which, in turn, cause the rhythmic release of Ach from MS neurons onto dentate cells (Lee et al., 2005; Zhang et al., 2010). Normally, those cholinergic signals would be filtered and processed in the dentate, and then conveyed through the CA fields, but when those signals arrive in the dentate of DPS hamsters, they hit a roadblock in the form of elevated synaptic inhibition. It seems likely that such a barrier would not only disrupt spatial memory encoding in the dentate-CA3 microcircuit, but would also disrupt the return signals from the hippocampus back to the septal complex, resulting in theta fragmentation. The microdialysis data further suggest that theta oscillations may be propagated along the septohippocampal pathway without concomitant release of Ach from septal neuronal terminals. This lack of Ach release is important because reductions in hippocampal Ach are a common cause of dementia (Teles-Grilo Ruivo and Mellor, 2013; Anand et al., 2017). Whether SCNx animals exhibit similar increases in synaptic inhibition or in cholinergic release is unknown, but their ability to compensate for theta fragmentation likely involves cholinergic signaling that is improved by increased cognitive effort.

I have purposely omitted any discussion of the medial prefrontal cortex (mPFC) in this model since it has not been addressed empirically in the hamsters. However, synchronous activity between the hippocampus and mPFC is critical to short-term spatial memory (Euston et al., 2012). The necessity of that interaction suggests that fragmented theta oscillations in septohippocampal circuits might convey misrepresentations of spatial information to the cortex, thus providing an additional locus of dysfunctional memory encoding. This model is just one way to think about the data. I have offered it—and done so with informal language—because my intention is to make the overall findings accessible to readers outside the fields of chronobiology and neuroscience. More detailed and formal presentations of these ideas may be found in the original research publications.

There is another facet of the DPS memory deficits that I think is important, but easily overlooked, and that is the severity of the memory deficits. It is clear that an active ARR SCN is capable of causing widespread dysfunction in the hippocampal formation. What is not immediately clear, is that memory deficits are so severe, that the circadian- ARR hamster is a distinct cognitive phenotype; an ARR SCN doesn’t just interfere with hippocampal memory, it obliterates it. The performance of DPS hamsters on memory tasks is not simply decreased by a few percent compared to controls; rather, they fail completely, never performing better than chance in the NOR and SA tasks. Even though we made the NOR task as easy as possible, with two very different objects in the test phase, they still failed, even when the time between the sample and test phases was only 20 min (Ruby et al., 2008). In the T-maze, DPS hamsters are unable to recall which arm of the T-maze they previously entered, even when that entry occurred only seconds earlier. In fear conditioning work currently underway, we are seeing the same phenomenon. Testing memory in ENT and DPS hamsters produces binary effects—one group passes, the other fails, with no middle ground.

## Conclusion

In humans, impaired cholinergic signaling is a hallmark of dementia and cognitive decline, which is accelerated by circadian dysfunction. The research summarized here suggests multiple loci where circadian rhythms and cognitive function intersect. The main benefit of the DPS model for translational studies is that it more closely resembles human circadian dysfunction than models employing SCN lesions or that manipulate genetics to alter SCN function. My colleagues and I were able to develop this model because we took an approach that was grounded in comparative physiology rather than in modern genetic techniques. The result is a simple method that uses light to break circadian timing in the SCN, but that also leaves the animals neurologically and genetically intact, all while the animals sit undisturbed in their home cages. In addition to the work we have done, other laboratories have used ARR Siberian hamsters to document impairments in social memory and object recognition (Müller et al., 2015; Müller and Weinert, 2016), as well as impairments in immune function (Prendergast et al., 2012). The DPS model can, in principle, be used to study the impact of circadian dysfunction on any physiological system or in the progression of disease and, unlike methods that employ constant reentrainment to phase-shifted LD cycles or LL to disrupt rhythms, we know that DPS animals do not incur any sleep loss (Larkin et al., 2004).

One of the goals of phototherapy is to shore up a weakened circadian system, a condition that is common among the aged. The DPS model might be valuable in achieving this goal. After all, if we can suppress circadian timing, why can’t we use targeted phototherapy to bring it back? This goal seems possible, particularly given some of the new phototherapeutic approaches under development (Najjar and Zeitzer, 2016; Negelspach et al., 2018; Kaladchibachi et al., 2019). In a number of unpublished failed experiments, I had no luck in restoring rhythms by daily melatonin injections (14 days), daily scheduled feeding (21 days), 2-deoxyglucose injections (to lower brain temperature and possibly reset the SCN), constant dark, and white light pulses. If a light treatment to restore circadian rhythms were developed in DPS animals, it would not only be of interest from a phototherapeutic perspective, but it would allow us to turn the SCN clock on and off with light. With such a system, one could essentially perform optogenetic experiments without surgery or any genomic modifications to the animals.

One of the reasons I turned to the field of learning and memory with the DPS model was that the neural circuitry that underlies so many memory tasks is well delineated. Unlike the SCN, output pathways from the hippocampus and related structures are well defined, so the task for the chronobiologist is to figure out how the SCN fits into those circuits. Having been in chronobiology for so many years, it still astounds me that we know so little about the functional outputs of the SCN. I consider it to be a gross oversight in our field because it continues to hold us back from better integrating into the medical community (Fernandez et al., 2021). We cannot claim a role for the circadian system in cognitive impairments such as Alzheimer’s disease and then shrug when asked how the SCN influences the hippocampus, or feel that it is sufficient to appeal to oscillator misalignment and expect that to satisfy medical professionals. The circuitry within the hippocampal formation is complex, but its connection to the SCN is not. Understanding how the SCN exerts control over the hippocampus is a manageable problem, one that will satisfy the goals of chronobiologists and clinicians alike.

## Author Contributions

NFR was solely responsible for this manuscript.

## Conflict of Interest

The author declares that the research was conducted in the absence of any commercial or financial relationships that could be construed as a potential conflict of interest.
